# Association between number of children and carotid intima-media thickness in Bangladesh

**DOI:** 10.1371/journal.pone.0208148

**Published:** 2018-11-27

**Authors:** Vylyny Chat, Fen Wu, Ryan T. Demmer, Faruque Parvez, Alauddin Ahmed, Mahbub Eunus, Rabiul Hasan, Jabun Nahar, Ishrat Shaheen, Golam Sarwar, Moise Desvarieux, Habibul Ahsan, Yu Chen

**Affiliations:** 1 Departments of Population Health and Environmental Medicine, New York University School of Medicine, New York, New York, United States of America; 2 Department of Epidemiology, Mailman School of Public Health, Columbia University, New York, New York, United States of America; 3 Department of Environmental Health Sciences, Mailman School of Public Health, Columbia University, New York, New York, United States of America; 4 U-Chicago Research Bangladesh, Ltd., Dhaka, Bangladesh; 5 INSERM UMR 1153, Centre de Recherche Epidemiologie et Statistique Paris Sorbonne Cité (CRESS), METHODS Core, Paris France; 6 Department of Health Studies, Center for Cancer Epidemiology and Prevention, The University of Chicago, Chicago, Illinois, United States of America; University of Rajshahi, BANGLADESH

## Abstract

Previous studies on the association between number of children and carotid intima-media thickness (cIMT) were limited to Western populations. Pregnancy in women is associated with physiologic changes that may influence the risk of cardiovascular disease. Comparing the association between number of children and cIMT in men and women can provide insights on whether the association may be due to pregnancy. We investigated the association between number of children and cIMT among 718 female (mean age 37.5 years) and 417 male participants (mean age 41.3 years), randomly selected from the Health Effect of Arsenic Longitudinal Study (HEALS), a population-based cohort study in Bangladesh. Multivariate linear regression was used to assess the association and to control for education attainment, history of diabetes, age, smoking, betel use, BMI, systolic blood pressure, and diastolic blood pressure. The average number of children was 4.43 for women and 3.74 for men. There were no nulliparous women. We observed a positive association between number of children and cIMT in women. Mean cIMT increased by 4.5 μm (95% CI, 0.8–8.1) per increment of one birth (P = 0.02). Compared to women with two children, cIMT in women with 4 children and ≥5 children was 23.6μm (95%CI, 2.6–44.7; P = 0.03) and 25.1 μm (95%CI, 3.5–46.6; P = 0.02) greater, respectively. The association was not modified by BMI, SBP, betel use or age. Data in men showed no evidence of association (P = 0.4). The finding suggests a role of high parity in atherosclerosis in women of a low-income, high parity population.

## Introduction

Cardiovascular disease (CVD) is the leading cause of death in women globally[1]. More CVD deaths are observed among women than men, specifically in low- and middle-income countries [2]. Studies have suggested that sex hormones may play a role in the etiology of CVD [3–6]. Pregnancy and childbirth are important life events for women that also significantly influence hormonal levels for sustained time periods. Mounting evidence has suggested association of parity with breast cancer, diabetes and obesity [7–9]. An emerging body of literature further extends these findings to CVD. Yet, available data remain equivocal, with positive associations found in two cross-sectional and two prospective studies [10–13]; a J-shaped association with two births as the nadir of risk was reported by a prospective cohort study [14]; and null associations were observed in a separate cross-sectional study and two other prospective studies [15–17]. Limited data are available informing possible pathological mechanisms despite the fact that such data can help to confirm or refute the hypothesis.

Carotid intima-media thickness (cIMT) is a reliable and non-invasive surrogate marker for atherosclerosis and has a direct dose-response relationship with incidence of CVD [18, 19]. cIMT can be measured among younger women prior to the development of clinical CVD disease. Several studies have investigated the relationship between parity and cIMT [20–25]. However, these studies generated inconsistent findings and only included Western populations consisting of large proportion of younger women with low parity (0–2 children) resulting in limited generalizability to developing countries where parity is high. We conducted a cross-sectional study assessing the association between number of children and cIMT among a subset of Bangladeshi women and men in the Health Effects of Arsenic Longitudinal Study (HEALS). The potential association between number of children and cIMT among women could be explained by both child-rearing and child-bearing. Therefore, we conducted analyses in men who were exposed to child-rearing but not childbearing.

## Methods

### Study population

Between October 2000 to May 2002, the Health Effects of Arsenic Longitudinal Study (HEALS), a population-based cohort study, began recruiting 11,746 men and women aged 18 years or more to study health effect of arsenic exposure in Araihazar, Bangladesh. Details of the study were included elsewhere [26]. HEALS is a valuable resource for examining other novel research topics due to its population-based study design, which involved door-to-door recruitment in an area that was no different from other rural areas in Bangladesh, along with comprehensive data collection on multiple demographic information, exposures and disease outcomes[27–32]. The study participants were sampled from a well-defined 25-km^2^ geographical area and all were married (to reduce loss to follow-up). This original cohort was further expanded during 2006–2008 to include 8,287 participants more (the expansion cohort) under the same criteria. The follow up activities took place at 2 year intervals with in-person physical examination and structured questionnaires. The majority of the study participants did not have formal education and were illiterate; verbal informed consent was obtained and recorded by trained interviewers and physicians. The procedures were approved by the Ethical Committee of the Bangladesh Medical Research Council and the Institutional Review Boards of Columbia University and University of Chicago.

Among the 11, 224 participants in the original cohort who provided urine samples at baseline and the 5,136 members in expansion cohort, we randomly selected 800 and 700 members who were over the age of 30 years old (because participants aged 30 years or less are not at appreciable risk of CVD), respectively, as part of our previous study on urinary arsenic and cIMT [28]. Of the total selected participants, 1,206 individuals completed cIMT measurements, and others did not, due to death, migration, illness or time constraint. Of the 1,206 members, 718 females and 417 males with complete number of children and clinical information are included presently. We have evaluated the effects of arsenic and anthropometric measures in a partially complete data [28, 29] and more recently effects of betel nut and periodontal disease [31, 33] in the same data.

### Questionnaire data

Information on demographic, lifestyle, and reproductive factors, including parity, defined as the total number of living and deceased children, was collected in a standardized questionnaire. Physicians used standard protocols and equipment to measure height, weight and blood pressures at baseline of the cohort, at follow-up, and at the time of cIMT measurement [34, 35]. In our study, we used data on body mass index (BMI), systolic blood pressure (SBP) and diastolic blood pressure (DBP) at cIMT measurement. Diabetes status at cohort baseline was defined by self-report of physician-diagnosed diabetes which was validated by comparison with results from glycosylated hemoglobin and glycosuria tests [36]. Smoking and betel nut chewing at cIMT measurement were dichotomized as “ever” or “never”.

### Assessment of cIMT

Carotid IMT was measured between April 2010 and September 2011, on average 6.6 years after the baseline of the parent HEALS cohort [31, 33]. Detailed methods of cIMT measurements were described elsewhere [28–31]. Briefly, we used a SonoSite MicroMaxx ultrasound machine (SonoSite, Inc., Bothell, WA, USA) equipped with an L38e/10-5-MHz transducer following the reading protocol implemented and validated in the Oral Infections and Vascular Disease Epidemiology Study (INVEST) [37]. A single designated physician with extensive experience performed the measurements blinded to the information on participants’ number of children. The INVEST protocol is conservative, minimizing scanning variability due to technical settings. cIMT data were analyzed offline with Matlab (Mathworks, Natick, MA), which calculated the mean and maximum of cIMT values automatically. We measured cIMT at both the near and far walls of the maximum common carotid artery cIMT from both sides of the neck. The mean of maximum measurements in 4 carotid sites was calculated in thousandths of a millimeter as it was found to be more associated with coronary atherosclerosis than an individual segment measurements [38].

### Statistical analysis

Descriptive statistics was conducted to assess difference in cIMT, demographic and clinical information by number of children using ANOVA for continuous variables and Chi-square tests for categorical variables. Multivariable linear regression was performed to investigate the association between number of children and cIMT. Number of children was treated as a continuous variable to estimate differences in cIMT associated with each additional child. To investigate non-linear relationships between number of children and cIMT, number of children was also categorized in five categories as 1, 2, 3, 4 or ≥ 5 children; individuals with five or more children were grouped together because the sample size in these separate categories was small. The first model was a crude model followed by the second model that was adjusted for risk factors of CVD or cIMT in the cohort, including educational attainment at baseline, age, smoking and betel use at cIMT measurement. In addition, BMI, history of diabetes, SBP, and DBP were further adjusted in the third model. These factors may be on the causal pathway between number of children and cIMT and therefore we included them in a separate model. Additional adjustment for HEALS baseline water arsenic, average urinary arsenic from baseline to the time of cIMT measurement, or age at first child birth did not make a difference (data not shown). For individuals with missing data (BMI, smoking, betel use, SBP, and DBP) measured at the time of cIMT assessment (n = 111–117 for women and n = 51–52 for men), we used the data ascertained at the most recent follow-up (2.4 years prior to cIMT measurement). A sensitivity analysis excluding participants with missing data was conducted and generated similar findings (data not shown). We also performed secondary analysis examining whether the association between dichotomized number of children (<4 vs. ≥4 children) and cIMT among women differed by other risk factors of atherosclerosis, with a cross-product term between number of children and either BMI (≤22 and >22 Kg/m^2^), SBP (≤120 and >120 mm Hg), betel use, or age (≤45 and > 45 years old). We dichotomized BMI, SBP and age using their mean values as cut-off points. Continuous number of children variable was rescaled to zero throughout the analysis as there were no nulliparous women in our study. Lastly, to evaluate the contribution of number of children to cIMT, we explored a structural equation model (SEM) with a simplified hypothesized path depicting only the associations between cIMT and independent variables of putative direct effects, including number of children, age at IMT, BMI, DBP, SBP, diabetes, and educational attainment. SPSS software (version 23, SPSS, Chicago, III) was used to perform all the statistical analyses except for SEM which was conducted using *lavaan* and *semPlot* packages in R 3.4.0.

## Results

The average number of total children was 4.43 (median 4 and range 1–13) among women, while men had slightly lower number of children (mean 3.74, median 3 and range 1–11). There were no nulliparous individuals in the study. The distribution of demographic, lifestyle, and clinical characteristics among women and men of different number of children was presented in Table 1. The mean of cIMT in the overall study population was 769.0 μm (±89.9 μm) for women and 825.0μm (±111.9 μm) for men. The average cIMT increased with number of children for both men and women (Table 1). The mean cIMT was 723.2 μm in women with 1–2 children, 759.9 μm in women with 3–4 children, and 797.6 μm in women with ≥5 children (P<0.001). For men, mean cIMT increased from 779.0 μm for those with 1–2 children to 823 μm for 3–4 children group to 873 μm for the ≥5 children category. The average year of formal education was 2.7 years in women and 3.6 years in men. Women with more children had a lower educational attainment and the same trend was observed in men (P<0.001). While the prevalence of betel nut use was comparable among women and men (50.4% vs. 52.0%, respectively), prevalence of smoking in men (74.3%) was more common than in women (9.5%). Individuals with more children were older, more likely to be ever smokers and betel nut users, tend to have a lower BMI and a higher SBP, compared to those with fewer children.

**Table 1:** Participant characteristics by numbers of children.

	Females					Males				
Characteristics	Overall (n = 718)	1–2 (n = 129)	3–4 (n = 290)	≥5 (n = 299)	P-value	Overall (n = 417)	1–2 (n = 125)	3–4 (n = 167)	≥5 (n = 125)	P-value
cIMT, μm (±SD)	769.0 ± 89.9	723.2 ± 73.4	759.9 ± 82.9	797.6 ± 93.1	<0.001	825.0 ± 111.9	779.0 ± 91.1	823.3 ± 115.6	873.3 ±106.5	<0.001
^a^Education, years(±SD)	2.7 ± 3.4	5.0 ± 4.0	2.8 ± 3.2	1.6 ± 2.6	<0.001	3.6± 4.0	4.6 ± 4.3	3.7 ± 4.0	2.5 ± 3.4	<0.001
^a^ History of Diabetes, %	2.2	0.8	1.7	3.3	0.19	1.4	1.6	1.2	1.6	0.95
^b^Ever betel using, %	50.4	27.9	43.8	66.6	<0.001	52.0	37.6	53.3	64.8	<0.001
^b^Ever smoking, %	9.5	3.9	6.6	14.7	<0.001	74.3	60.0	77.2	84.8	<0.001
^b^BMI, Kg/m^2^(±SD)	21.5 ± 4.2	23.4 ± 4.6	21.7 ± 4.3	20.4 ± 3.7	<0.001	20.2 ± 4.3	21.2 ± 5.3	20.2 ± 3.6	19.3 ± 4.0	<0.001
^b^Diastolic blood pressure, mm Hg	75.9 ± 11.8	76.7 ± 12.1	75.3 ± 11.5	76.2 ± 12.0	0.43	76.2 ± 12.2	75.4 ± 12.6	76.1 ± 11.4	77.1 ± 12.7	0.52
^b^Systolic blood pressure, mmHg	120.0 ± 16.4	119.3 ± 16.3	118.4 ± 15.2	121.9 ± 17.4	0.03	121.4 ± 16.1	118.7 ± 15.5	121.5 ± 14.8	124.0 ± 17.9	0.03
^b^Age, years	44.2 ± 8.5	37.1 ± 6.5	41.8 ± 6.9	49.5 ± 7.3	<0.001	48.1 ± 9.4	40.9 ± 6.4	47.8 ± 7.7	55.6 ± 8.2	<0.001

^a^ Measurements at baseline,

^b^ Measurement at cIMT

For women, there was a statistically significant positive association between number of children and cIMT in all models (Table 2). After adjusting for educational attainment, age, smoking and betel nut use, the association remained statistically significant (P = 0.02 when number of children was considered as a continuous variable). Model 2 further controlled for remaining potential mediators including BMI, SBP, DBP and history of diabetes. The association remained similar and significant (P = 0.02), with a cIMT increase of 4.5 μm (95%CI: 0.8, 8.1) per additional birth. Analyses modeling number of children categorically suggested the nadir of risk was among women with 2 children. Compared with women who had two children, cIMT level did not differ significantly in women with one or three children but was lower than women with 4 or ≥ 5 children. In the final model, compared to the reference group of women with 2 children, women with 4 and at least 5 children had a significantly higher cIMT level (P = 0.03 and 0.02 respectively).

**Table 2:** Association between number of children and cIMT.

	Number of Children					
	1	2	3	4	≥5	Number of Children as a cont. variable
**Females**						
N	30	99	137	153	299	718
** Crude Model**						
Expected β (95%CI)	-3.9 (-39.0,31.2)	**Ref**	29.3 (7.1,51.6)	41.4 (19.7,63.1)	73.6 (54.2,93.0)	14.3 (11.4,17.3)
P value	0.8		0.01	<0.001	<0.001	<0.001
** Model 1**^a^						
Expected β (95%CI)	3.6 (-29.6,36.4)	**Ref**	16.2 (-4.8,37.3)	22.1 (1.0,43.2)	23.7 (2.3,45.1)	4.3 (0.7,8.0)
P value	0.8		0.1	0.04	0.03	0.02
** Model 2**^b^						
Expected β (95%CI)	5.9 (-26.9,38.7)	**Ref**	17.9 (-3.1,38.9)	23.6 (2.6,44.7)	25.1 (3.5,46.6)	4.5 (0.8,8.1)
P value	0.7		0.1	0.03	0.02	0.02
**Males**						
N	38	87	88	79	125	417
** Crude Model**						
Expected β (95%CI)	-38.2 (-78.6,2.3)	**Ref**	30.9 (-0.6,62.3)	34.7 (2.4,67.1)	82.6 (53.6,111.7)	20.4 (15.2,25.6)
P value	0.06		0.05	0.03	<0.001	<0.001
** Model 1**^a^						
Expected β (95%CI)	-20.5 (-57.8,16.8)	**Ref**	9.0 (-20.0,38.0)	-12.9 (-44.3,18.4)	3.4 (-29.0,35.8)	1.4 (-5.4,8.2)
P value	0.3		0.5	0.4	0.2	0.7
** Model 2**^b^						
Expected β (95%CI)	-20.6 (-56.7,15.5)	**Ref**	14.2 (-13.9,42.3)	-12.4 (-43.0,18.2)	7.7 (-24.1,39.4)	2.8 (-3.8,9.4)
P value	0.3		0.3	0.4	0.6	0.4

^a^ Adjusted for education attainment, age, smoking and betel use;

^**b**^ Model 1^a^ + history of diabetes+ BMI + SBP + DBP (at cIMT)

For men, however, the association was not apparent after adjusting for potential confounders (Table 2), either when number of children was treated as continuous or categorical variable. Similarly, no noticeable trend was observed for the association between number of children and cIMT across categories in the adjusted models in men.

In women, we further tested for interaction between several characteristics with number of children (S1 Fig), adjusted for multiple covariates in the final model of linear regression analysis. Number of children was dichotomized into <4 and ≥4 children in these analyses. The association between number of children and cIMT did not differ by BMI, SBP, betel nut use or age. In SEM, age at IMT was the strongest predictor of cIMT in women, followed by number of children and other variables. For every one standard deviation increase in age, cIMT increases by 0.38 standard-deviation (p-value<0.001), while one standard-deviation increment in total births was associated with 0.11 standard-deviation increase in cIMT (p-value = 0.01). The standardized effects of other variables were smaller, ranging from -0.01to 0.07 (data not shown).

## Discussion

In our study, we found a positive association between number of children and cIMT among parous women. After adjusting for educational attainment and history of diabetes, age, smoking, betel use, BMI, SBP and DBP, each additional birth was related to a difference of 4.5 μm in cIMT (95%CI: 0.8,8.1). Categorizing number of children into five categories revealed the nadir level of risk to be among women with two children. The association was most marked for women with four or more children. Interestingly, the observed significant association was limited to only women and not men. Tests for heterogeneity across demographic and clinical characteristics (BMI, SBP, betel use and age) demonstrated no evidence of effect modification in the association of number of children and cIMT among women.

### The association between number of children and cIMT in women

In our final model after adjusting for potential confounders, cIMT was 23.6 μm (P = 0.03) and 25.1 μm (P = 0.02) greater in women with four and five or more children, respectively, compared with women who had two children. Previous studies reported that a difference of 100μm cIMT among women can be translated into a 35% increased incidence of coronary heart disease (CHD) [39]. Thus, our findings suggest roughly an increase in CHD incidence by 8–9% for women with four and five or more children compared with women who had two children. In our study population, about 63% of women have at least 4 children. The findings may have public health impact for CVD prevention and risk assessment in Bangladesh and other countries where high parity is common.

Our findings suggest a positive association between high parity and cIMT in women. To the best of our knowledge, there were six prior studies investigating the association between parity and cIMT among women (S1 Table). Four of them reported consistent results suggesting a positive association between parity and cIMT. However, these studies were conducted among European women of limited parity number (38–89% of participants with parity <2) [20–22]. In Finnish women, per increment of one birth was associated with a difference of 6.4 μm in cIMT (P = 0.05). A study conducted in the Netherland found a positive trend of higher levels of mean cIMTs among women with 0, 1, 2–3 and ≥ 4 children [21]. The adjusted mean cIMTs for each parity category were 750, 740, 770 and 810 (μm), respectively (P for trend = 0.005). A cross-sectional study of high-risk women with at least one CVD risk factor in France also indicated a higher level of cIMT by 7μm for every increment in parity (p = 0.056) [22]. Lastly, categorical data of parity in a cross-sectional German study described the association as U-shaped, in which women with 1–2 children had the lowest mean cIMT [24]. Similar to our findings, high mean cIMT was observed to be most pronounced among women of highest parity (β = 0.77 mm; 95%CI = 0.74–0.80), though in the present study we only included parous women.

Two studies reported contradicting results; a null association was found (S1 Table). The first one, a Finnish cross-sectional study, demonstrated a positive association only in crude model. After adjustment for potential confounders, the association attenuated [23]. The absence of association in the Finnish study could be explained by the limited number of women of high parity. The highest parity group was ≥ 4, accounting for only 9.7% of the study population. The US study similarly did not discover any statistically significant difference in cIMT across parity categories (P = 0.062), after adjusting for age and race [25]. It should be noted that the US study was conducted among women of BMI of 25–39.9 Kg/m^2^. In our study, all women were parous, and unlike the Finnish study, we had a large sample size of women with high parity and an increased power to detect an association. Our population is relatively lean (mean BMI = 22 Kg/m^2^) and those with a BMI > 22 Kg/m^2^ may still be at the low end of BMI in Western populations. Lean women may be more susceptible to the effect of parity on cIMT; albeit more studies are needed to confirm the hypothesis. Our exploratory analyses using SEM also suggest number of children is a critical contributor to cIMT in women, secondary to age. Taken together, the existing studies on parity and cIMT, although limited, are consistent with a positive association between high parity (four and above) and cIMT in Western populations as well as in low-income and Asian populations, suggesting a role of high parity in atherosclerosis.

### The association between number of children with cIMT among men

Results in men indicated no association between number of children and cIMT. This finding suggests a limited role of child rearing (social, lifestyle factors and family size) in increased atherosclerosis risk. There were only two previous studies investigating the association among men. A study following a Finnish cohort for cIMT change during 6 years period reported a negative association between number of children and cIMT among men [20]. More men in this study had number of children at the lower end (64% nulliparous men) and the highest number of children category was ≥2. However, our study’s design and population are more comparable with a cross-sectional study by Skilton and colleagues, which reported a null association, consistent with our finding [22].

Given the lack of association among men, the likelihood that the adverse effect of number of children on risk of CVD in women is due to child rearing is reduced. However, we cannot account for child-rearing associated factors that differ between males and females. Child bearing or pregnancy may lead to a direct physiological effect. To nurture the development of the fetal brains, fatty acids are required. As a response, mobilization of fat from the lower body parts to the abdomen occurs [40]. A study conducted among African American and White young adults reported abdominal fat accumulation could contribute to increased atherosclerosis progression [41]. Pregnancy is also associated with insulin resistance. The increased insulin insensitivity associated with pregnancy may stimulate high circulatory fatty acids and secretion of VLDL (very low density lipid) by the liver, resulting in enhanced risks of CVD among parous women [42]. Increase in oxidative stress associated with pregnancy may lead to increased level of lipid peroxidation and inflammatory responses that contribute to endothelial dysfunction and atherogenesis [43, 44]. The repeated exposures to these pregnancy-related changes, as a result of high parity, may lead to an elevated level of atherosclerosis, and such influences may have a greater cumulative impact later in life when women are more susceptible to CVD. Previous studies also reported an association between post-menopause with increased mean cIMT and CVD risks, which could be due to old age or hormonal change [4, 5, 45].

Our study has certain strengths. The participants were randomly recruited from a large population-based cohort study. The cIMT measurements were conducted under standardized protocols by an experienced physician blinded to the exposure variables to minimize measurement errors. Also, our population has a wide range of number of children, allowing investigation of its effect on cIMT at the high end. cIMT was employed as a marker for atherosclerosis; thus, we could observe the proximal impact of number of children on physiological changes relevant to CVD before occurrence of clinical endpoints. Unlike the study by Skilton and colleagues [22], we did not restrict the participants to high-risk women; the generalizability of our findings is increased. Moreover, with data from men as a control group, we were able to differentiate whether the association between number of children and cIMT was mainly due to pregnancy itself (child bearing) or behaviors associated with child rearing.

There are several potential limitations. Our data on number of children were collected through questionnaires subjecting the information to recall bias. Yet, it is unlikely that participants would report differently on their reproductive history based on their unknown cIMT measurements. Similarly, the information was collected only at the baseline of parent HEALS study. During the window period between baseline and IMT assessment, women, and particularly younger women could have more children. The potential non-differential exposure misclassification would have resulted in the attenuation of the observed positive association between number of children and cIMT in our study. Data on other risk factors of CVD such as gestational diabetes, preeclampsia and insulin resistance were not collected. However, these factors are more likely to be resulted from parity making them potential mediators in the causal pathways of parity and CVD, rather than potential confounders.

## Conclusion

Our study is the first to investigate the association between number of children and cIMT, a non-invasive subclinical marker for atherosclerosis, among South Asian women. We found a positive association between number of children and cIMT among women but not men. As such, the results indicate a role of high parity in atherosclerosis and the development of CVD among parous women. Our findings warrant the consideration of reproductive history in primary prevention for CVD in women with low income, high parity and shared characteristics with our study population.

## Supporting information

S1 Fig Effect of number of children on cIMT among females. We investigated potential effect modifiers and plotted β coefficient estimates for each strata using a forest plot. The association between number of children and cIMT among females was not modified by BMI, SBP, betel use or age (p for interaction >0.05).
(DOCX)

S1 Table Association between number of children and IMT in previous studies. Here you find a list of previous studies on the associations between number of children and cIMT. The table included characteristics of the study populations, covariates adjusted in the analyses as well as main findings of the studies.
(DOCX)
