# Supplementary material for: Association between number of children and carotid intima-media thickness in Bangladesh
Source: PLoS One. 2018 Nov 27;13(11):e0208148. doi: 10.1371/journal.pone.0208148 (PMC6258552; doi:10.1371/journal.pone.0208148)
Supplement: S1 Fig — We investigated potential effect modifiers and plotted β coefficient estimates for each strata using a forest plot. The association between number of children and cIMT among females was not modified by BMI, SBP, betel use or age (p for interaction >0.05). (DOCX) [file pone.0208148.s001.docx]

**S1 Fig: Effect of Number of Children on cIMT among Females ^a^**

**Potential Effect Modifiers (N)**

BMI

≤ 22 (n=437)

> 22 (n=281)

SBP

≤ 120 (n=494)

> 120 (n=224)

Betel Using

No (n=356)

Yes (n=362)

Age

≤ 45 (n=426)

> 45 (n=292)

**β coefficient**

0.6

0.5

0.5

0.7

**P-value for interaction**

**P-value**

0.1

0.3

0.03

0.9

0.2

0.2

0.07

0.2

**β coefficient (95%CI) ^b^**

15.5 (-3.9, 35.0)

10.9 (-9.5, 31.3)

17.5 (1.4, 33.6)*

0.8 (-27.2, 28.8)

12.6 (-5.9, 31.0)

13.7 (-7.9, 35.4)

13.6 (-1.2, 28.3)

23.1 (-8.5, 54.7)

^a^ Number of children were dichotomized into parity <4 (N=266) and parity ≥4 (N=452)

^b^ Adjusted for education attainment, history of diabetes, age, smoking, betel, BMI, SBP and DBP at IMT measurements

*Significant at P<0.05
