# Supplementary material for: Association between number of children and carotid intima-media thickness in Bangladesh
Source: PLoS One. 2018 Nov 27;13(11):e0208148. doi: 10.1371/journal.pone.0208148 (PMC6258552; doi:10.1371/journal.pone.0208148)
Supplement: S1 Table — Here you find a list of previous studies on the associations between number of children and cIMT. The table included characteristics of the study populations, covariates adjusted in the analyses as well as main findings of the studies. (DOCX) [file pone.0208148.s002.docx]

**S1 Table: Association between Number of Children and cIMT in Previous Studies**

| **First Author (Country)** | **Study Population** | **N** | **Mean Age  in Years** | **Number of children** | **Adjusted for** | **Findings** |
| --- | --- | --- | --- | --- | --- | --- |
| Skilton 2010 (Finland) [[1](#_ENREF_1)] | Prospective cohort study with Finnish participants followed from 2001-2007 | 1,005 females  781 males | 28-33 | Childbirth within 6 year follow-up  0  1  ≥ 2 | Age, baseline no. children, cIMT, employment status, marital status, cardiovascular risk factors at baseline and at the end of study | Mean cIMT increased by 6.4µm per child born (p=0.05) for women. Negative association was observed among males (P trend= 0.04). |
| Humphries 2001(The Netherlands) [[2](#_ENREF_2)] | Cross-sectional study in Ommoord, a suburb of Rotterdam, Netherland | 4,878 females | 55-99 | 0  1  2-3  ≥ 4 | Age, smoking, socioeconomic, hypertension, diastolic and systolic blood pressures, BMI, lipids and insulin/glucose ratio at baseline | Adjusted mean IMTs were 750, 740, 770 and 810 (µm) for women in each parity categories respectively. There was a statistically significant positive trend (p=0.005). |
| Skilton 2009 (France) [[3](#_ENREF_3)] | Cross-sectional study of outpatients at the Centre for Prevention and Detection of Atherosclerosis | 718 females  1,164 males | 18-80 | 0  1  2-3  ≥ 4 | Age, employment, marital status, physical activity, waist circumference, dietary, score, smoking, hypertension, HDL cholesterol, LDL cholesterol, log triglycerides and glucose. | Per increment of one child was related to a difference of 7 µm in mean cIMT (p=0.056). The difference in IMT only marked among women of ≥ 4 births compared to nulliparous women (β=62 µm, p=0.006). Among men, the association was not significant (p=0.74). |
| Kharazmi 2007 (Finland) [[4](#_ENREF_4)] | Cross-sectional study of the nationally representative population of Finns | 746  females | 45-74 | 0  1  2-3  ≥ 4 | Age, systolic and diastolic blood pressure, fasting blood glucose and cholesterol, education, smoking and body mass index | The association between cIMT and parity was significant only in crude model. Further adjustment for age and other covariates removed the significant association (p=0.48). |
| Wolff 2005 (Germany)[[5](#_ENREF_5)] | Cross-sectional study from Population in West Pomerania | 1,195 females | 20-79 | 0  1  2  3  ≥ 4 | Age, socioeconomic factors, lifestyle variables, hypertension, hormone replacement therapy, age at menopause and oral contraceptives | There was a U-shaped association between mean cIMT and parity. Women with 1 and 2 children had the lowest mean IMT compared to nulliparous and multiparous women. |
| Niemczyk 2015 (USA)[[6](#_ENREF_6)] | Secondary analysis data of participants from the Slow Adverse Vascular Effects (SAVE) clinical trial in Allegheny Country, PA (overweight/obese women with BMI 25-39.9 kg/m^2^) | 172  females | 25-45 | 0  1  2  ≥3 | Age, race, BMI, education, current smoking, alcohol, average systolic blood pressure and fasting glucose level on the carotid measures | Mean cIMT values did not differ by parity. Yet, when categorized as nulliparous and parous women, there was a statistically significant negative association. Nulliparous women had thicker CCA-IMT than parous women (p=0.007) by 29 µm (p=0.009). |

**References**

1. Skilton MR, Bonnet F, Begg LM, Juonala M, Kähönen M, Lehtimäki T, et al. Childbearing, child-rearing, cardiovascular risk factors, and progression of carotid intima-media thickness. Stroke. 2010;41(7):1332-7.

2. Humphries KH, Westendorp IC, Bots ML, Spinelli JJ, Carere RG, Hofman A, et al. Parity and carotid artery atherosclerosis in elderly women. Stroke. 2001;32(10):2259-64.

3. Skilton MR, Sérusclat A, Begg LM, Moulin P, Bonnet F. Parity and Carotid Atherosclerosis in Men and Women. Stroke. 2009;40(4):1152-7.

4. Kharazmi E, Moilanen L, Fallah M, Kaaja R, Kattainen A, Kähönen M, et al. Reproductive history and carotid intima-media thickness. Acta obstetricia et gynecologica Scandinavica. 2007;86(8):995-1002.

5. Wolff B, Völzke H, Robinson D, Schwahn C, Lüdemann J, Kessler C, et al. Relation of parity with common carotid intima-media thickness among women of the study of health in Pomerania. Stroke. 2005;36(5):938-43.

6. Niemczyk NA, Catov JM, Barinas‐Mitchell E, McClure CK, Roberts JM, Tepper PG, et al. Nulliparity is associated with less healthy markers of subclinical cardiovascular disease in young women with overweight and obesity. Obesity. 2015;23(5):1085-91.
